# 

**Published:** 1908-01

**Authors:** 


### CONTENTS.

ORIGINAL COMMUNICATIONS—
How to Prevent the Spread of Contagious Diseases. By Samuel A. Visanska, Ph. G., M. D., Atlanta, Georgia .......... 575
Typhoid Fever. By W. A. Mathews, M. D. Hawkinsville, Georgia .......... 583
Strangulated Inguinal Hernia in Infants With Report of a Case. By John Egerton Cannaday, M. D., Hansford, West Virginia .......... 589
Perforating Ulcer of the Foot. By Dr. M. G. Campbell, Atlanta, Georgia .......... 593
DISCUSSION .......... 597
A Review of the Modern Treatment of Gonorrhea. By C. W. Canan, M. D., B. S., Oakley Springs, Virginia .......... 598
Medicinal Effects of Indian Springs Water on Typhoid Fever. By A. F. White, M. D. Flovilla, Ga. .......... 600

EDITORIAL NOTES AND COMMENTS—
Ward Teaching .......... 603

Rose Water .......... 605

NEWS AND NOTES .......... 606

BOOK REVIEWS .......... 614

SELECTIONS AND ABSTRACTS—
The Small Medical Colleges .......... 616
Technic of Stump Inversion in Appendectomy .......... 617
The Duty of the Physician to the School Child .......... 618
Alexander Mactier Pirrie .......... 619
Independence in Medical Journalism .......... 621
Medical Ethics .......... 622
Colorado and the Eastern Pulmonary Case .......... 624
Dermatological Ideas .......... 626
Peritonitis and its Treatment .......... 627
International Congress on Tuberculosis .......... 630
MEDICAL ITEMS— .......... 636

